# Heme interaction of the intrinsically disordered N-terminal peptide segment of human cystathionine-β-synthase

**DOI:** 10.1038/s41598-018-20841-z

**Published:** 2018-02-06

**Authors:** Amit Kumar, Amelie Wißbrock, Nishit Goradia, Peter Bellstedt, Ramadurai Ramachandran, Diana Imhof, Oliver Ohlenschläger

**Affiliations:** 1 Leibniz Institute on Aging – Fritz Lipmann Institute, Beutenbergstr. 11, D-07745 Jena, Germany; 2 Pharmaceutical Biochemistry and Bioanalytics, Pharmaceutical Institute, University of Bonn, An der Immenburg 4, D-53121 Bonn, Germany; 3 Friedrich Schiller University, Faculty of Chemistry and Earth Sciences, Humboldtstr. 10, D-07743 Jena, Germany

## Abstract

Cystathionine-β-synthase (CBS) belongs to a large family of pyridoxal 5’-phosphate (PLP)-dependent enzymes, responsible for the sulfur metabolism. The heme-dependent protein CBS is part of regulatory pathways also involving the gasotransmitter hydrogen sulfide. Malfunction of CBS can lead to pathologic conditions like cancer, cardiovascular and neurodegenerative disorders. Truncation of residues 1–40, absent in X-ray structures of CBS, reduces but does not abolish the activity of the enzyme. Here we report the NMR resonance assignment and heme interaction studies for the N-terminal peptide stretch of CBS. We present NMR-spectral evidence that residues 1–40 constitute an intrinsically disordered region in CBS and interact with heme *via* a cysteine-proline based motif.

## Introduction

Cystathionine-β-synthase (CBS) is a modular enzymatic protein of 551 amino acids. The lyase CBS acts in the transsulfuration pathway and has a central role in the mammalian sulfur metabolism by catalysing the condensation of serine and homocysteine to a cystathionine intermediate^1,2^. Mutations in CBS are responsible for an inborn autosomal recessive inherited deviation in the amino acid metabolism, homocystinuria. This rare disease results in a clinical phenotype with elevated levels of homocysteine in blood plasma affecting four central organ systems, the cardiovascular, ocular, skeletal, and central nervous system and finally manifesting e.g. in neural tube defects, cardiovascular diseases and Alzheimer’s disease^3–5^. Currently, more than 150 pathogenic CBS mutations have been reported which are spread across the coding sequence^6,7^. CBS is a heme-binding protein and represents an example for a human hemoprotein that can be regulated by gases *via* their heme component^8^. Especially, besides its binding CO and NO^9^, CBS is involved in cellular production of major amounts of the gasotransmitter H_2_S^10^. It was shown that aberrant function of CBS may thus lead to cancer, cardiovascular and neurodegenerative diseases making it an interesting drug target^11–13^.

**Figure 1:**
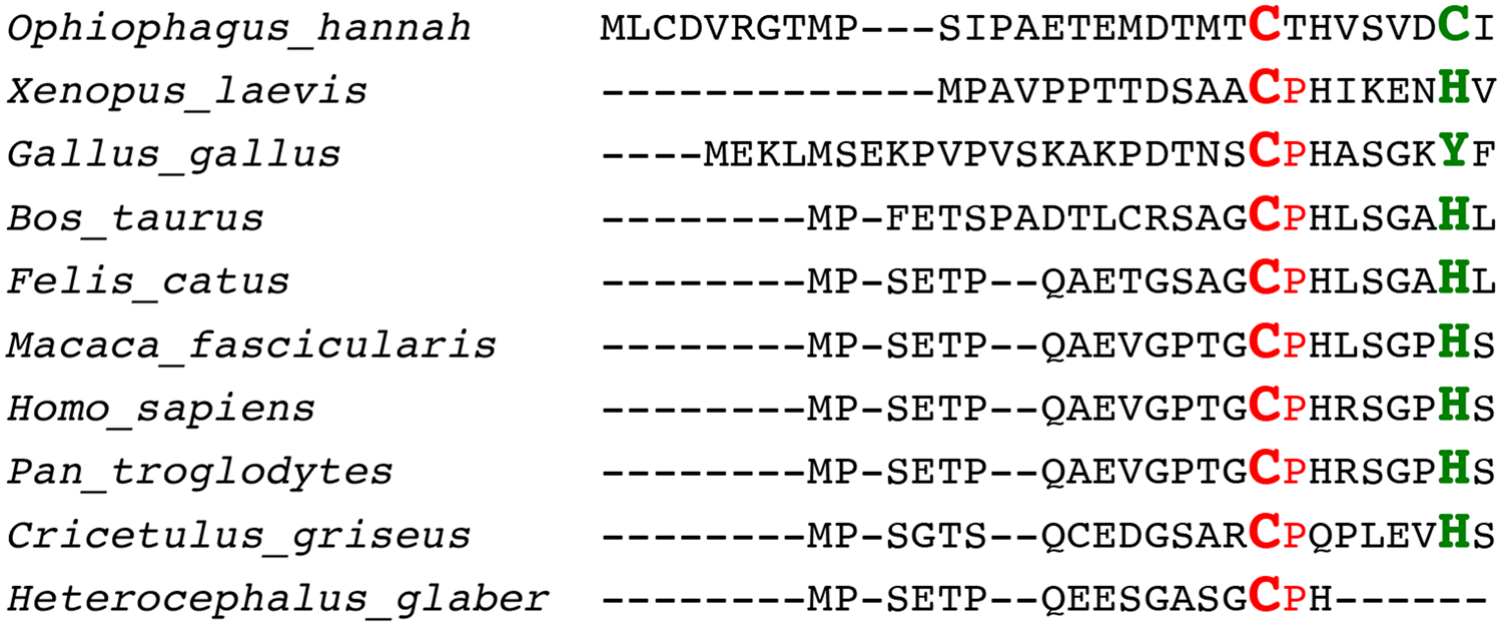
Sequence alignment of the N-terminal stretches of CBS proteins from different organisms.

The homotetrameric CBS, a member of the fold type II family of the pyridoxal 5′-phosphate (PLP)-dependent enzymes, is in large parts structurally well characterised^14,15^. Members of this enzyme family are of multiple evolutionary origins. CBS contains the N-terminal heme-binding domain followed by catalytic core, which is conserved in most proteins of this class of organisms, and a C-terminal regulatory domain. The N-terminal domain (residues 1–70) is sequentially diverse in many classes of organisms and absent in lower eukaryotes like yeast and mycobacterium. The canonical heme in CBS is bound *via* an axial coordination to cysteine-52 and histidine-65 in the N-terminal region of the molecule. Interestingly, this binding site is missing in the homologous yeast enzyme^14,16^. In contrast to covalent binding of heme, recent studies have shown that transient interactions of the heme moiety with proteins can act as functional triggers^17^. Here, heme-binding motifs (HBM) or heme regulatory motifs (HRM) with special amino acid combinations –among them the cysteine-proline (CP) motif– are suggested to be responsible for heme association^17–22^. However, due to moderate binding constants these motifs at the same time allow also a fast dissociation of the heme complex. Interestingly, also in the newest X-ray structures of the human CBS protein^23,24^ (e.g. PDB code 4COO) the stretch from residue 516–526 and the first 42 N-terminal residues are missing. Exactly in the latter region a CP-motif is located which is followed by histidines at positions *P* + *2* and *P* + *7* relative to the cysteine. This region is structurally close to the canonical heme-binding site at Cys^52^/His^65^ which allows to speculate about a scavenger-type function of the CP-motif located in this intrinsically disordered protein region to support the localisation of the heme molecule during the binding process or to act as a second independent heme-binding site. In addition, it might be of interest to note that a sequence homology analysis reveals this CPH-motif to be conserved in higher eukaryotes with secondary interacting amino acid (H, C, Y) present in vicinity (Fig. 1).

Intrinsically disordered protein regions (IDPRs) are increasingly recognised to have functional relevance for biological regulatory processes in e.g. cellular signal transduction, molecular recognition and transcription^21,25,26^ making them an interesting target for drug intervention^27,28^. Bioinformatic predictions (e.g. with DISOPRED2^29^) estimate that disordered regions or segments longer than 30 residues are present in approx. 2.0% of archaean, 4.2% of eubacterial and 33.0% of eukaryotic proteins^30^ and that IDPRs are highly abundant in disease-related proteins^31^. Meanwhile the aim to structurally characterise IDPRs has fostered the development of various approaches e.g. in the areas of computational prediction^30,32,33^ as well as NMR spectroscopy^34–43^. Interestingly, CP-motifs are also found in intrinsically disordered regions of functional relevance in other proteins that are associated with heme binding^21^.

In this context, we have expressed the CBS protein using established approaches and investigated the heme-binding domain separately from the catalytic core. Functional studies on CBS(1–413) and on mutations of the non-canonical heme-binding site (C15S) were performed to compare and analyse the heme-binding process in CBS. NMR spectroscopy was also employed to characterise the heme interaction exploiting the advantage of this technique to describe also intrinsically disordered regions at atomic level.

## Results

### Protein expression and purification

The recombinant CBS(1–413) and the CBS(1–413)C15S mutant were expressed and purified as per literature^44^. The N-terminal CBS peptide (_GB1_CBS(1–40)), consisting of the residues 1–40 of CBS (MPSETPQAEVGPTGCPHRSGPHSAKGSLEKGSPEDKEAKE), was expressed as fusion to the B1 domain of streptococcal protein G (GB1)^45^.

### UV/Vis study

**Figure 2:**
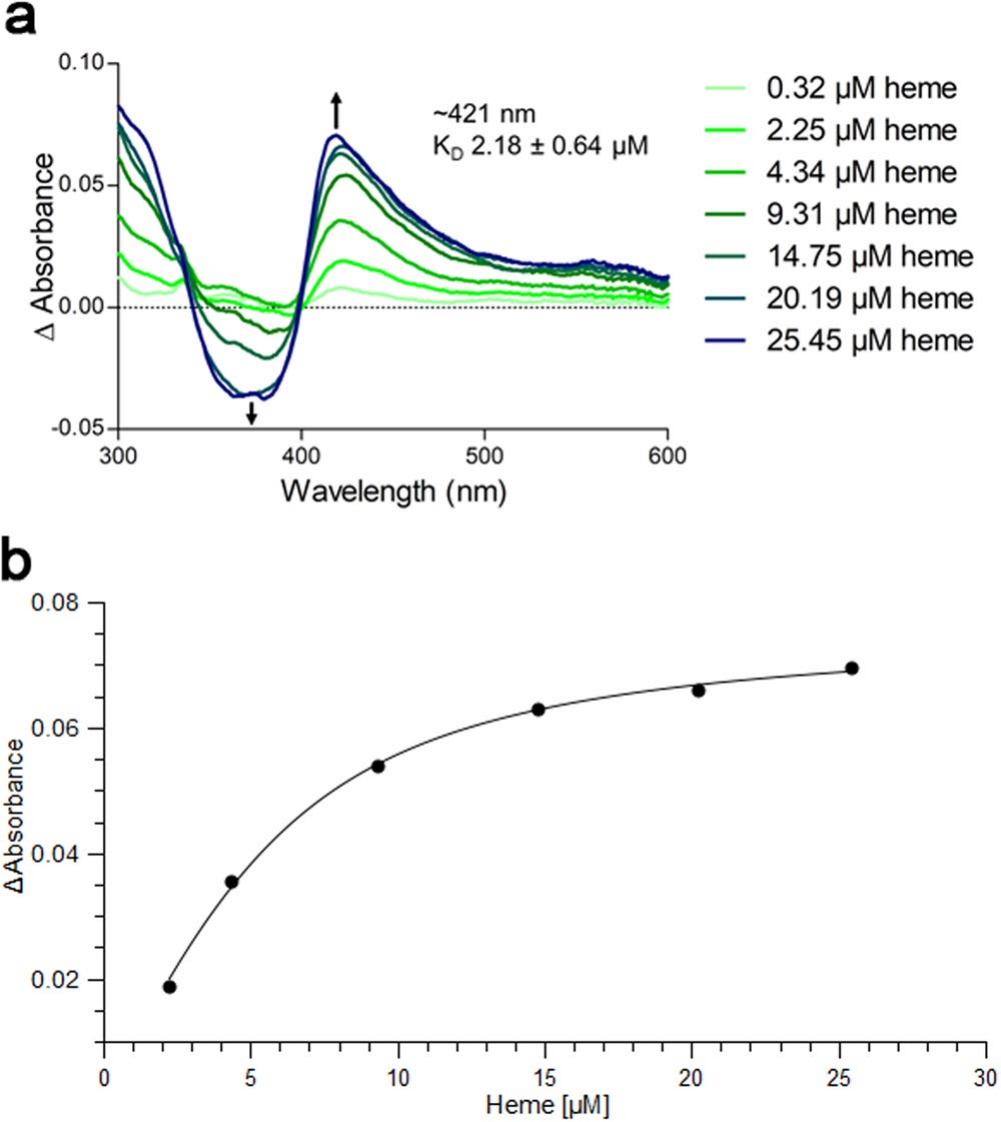
Heme binding to the _GB1_CBS(1–40) fusion protein determined by UV/Vis spectroscopy. (**a**) The _GB1_CBS(1–40) fusion protein was incubated with hemin at the concentrations indicated (5 μM protein, 0.32–25.45 μM hemin). (**b**) Titration curve observed at 421 nm.

UV/Vis spectroscopy was employed to monitor the interaction of the proteins with heme. UV/Vis data shows the heme binding by a Soret band shift to ~421 nm (Fig. 2a). Previous studies on heme binding to peptides using UV/Vis, Raman, EPR and NMR spectroscopy revealed that a UV shift to ~420 nm may be related to a hexacoordinated complex^46–50^. Thus, this bathochromic effect is characteristic of heme binding and suggests formation of a hexacoordinated state which in CBS involves residues cysteine-15 and histidine-22. The K_D_ value was determined to be 2.18 ± 0.64 μM. According to the best fit possible _GB1_CBS(1–40) binds to heme with a 1:1 stoichiometry (Fig. 2b).

### Enzyme activity assay

**Figure 3:**
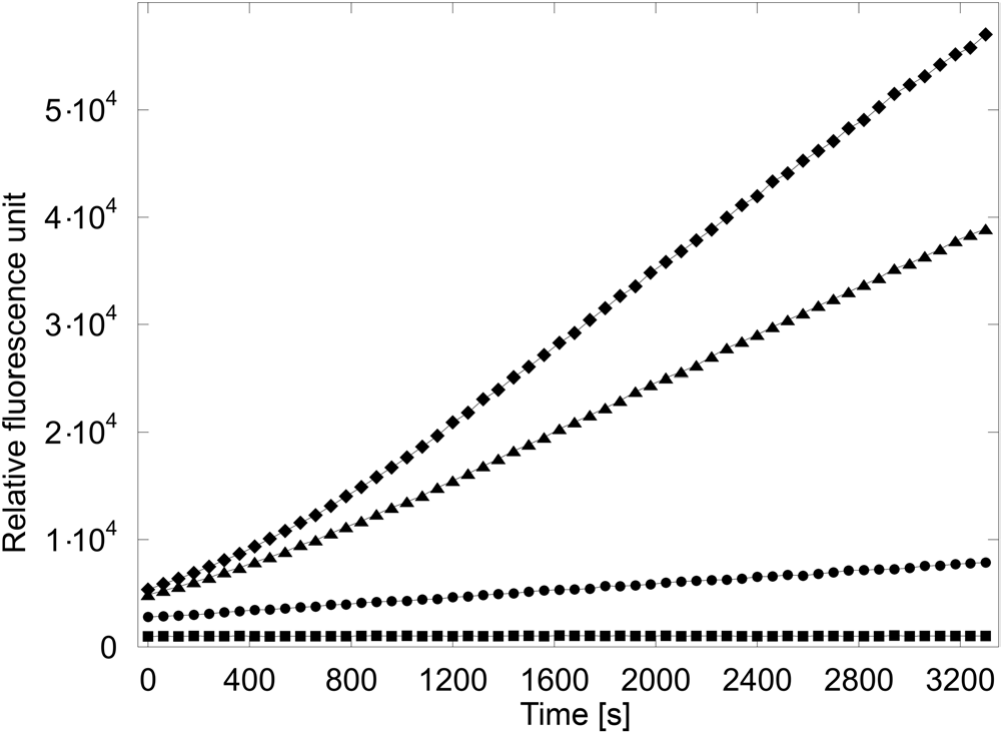
Fluorescence assay of CBS(1–413) and CBS(1–413)C15S. The average curve of six independent measurements is displayed. Symbols used: ◆ = CBS(1–413), ▲ = CBS(1–413)C15S, ● = blank, no protein, ■ = blank, no probe.

The relative activities of CBS(1–413) and of the CBS(1–413)C15S mutant were compared in an enzyme activity assay using 7-azido-4-methylcoumarin as probe as described earlier in the literature^10^. To assure reproducibility six independent measurements were performed. During expression of the two enzymes in *E. coli* heme is endogenously produced and incorporated in the CBS proteins. This is reflected in yellowish colours of the protein samples with a darker colour of the CBS(1–413) with respect to the CBS(1–413)C15S mutant (data not shown) as shown for CBS and other recombinant heme-binding molecules^51,52^. Hence, exogenous addition of heme during the assay does not lead to activity differences off to the mean from the independent measurements of both CBS(1–413) and CBS(1–413)C15S mutant. This indicates the saturation of the compounds with endogenous heme already during expression in the *E. coli* cells. Figure 3 shows that CBS(1–413)C15S was on average 32% (between 20% to 40% in the individual measurements) less active than CBS(1–413). The assay indicates that beside of its canonical binding site at Cys^52^/His^65^ heme is also binding to the CP-motif at position 15 and involving H22. This result of the assay is consistent with an observation of a previous study^44^ where truncation of amino acids M1-K39 in CBS led to a similar decrease in enzyme activity.

### NMR resonance assignment of the CBS N-terminal peptide

**Figure 4:**
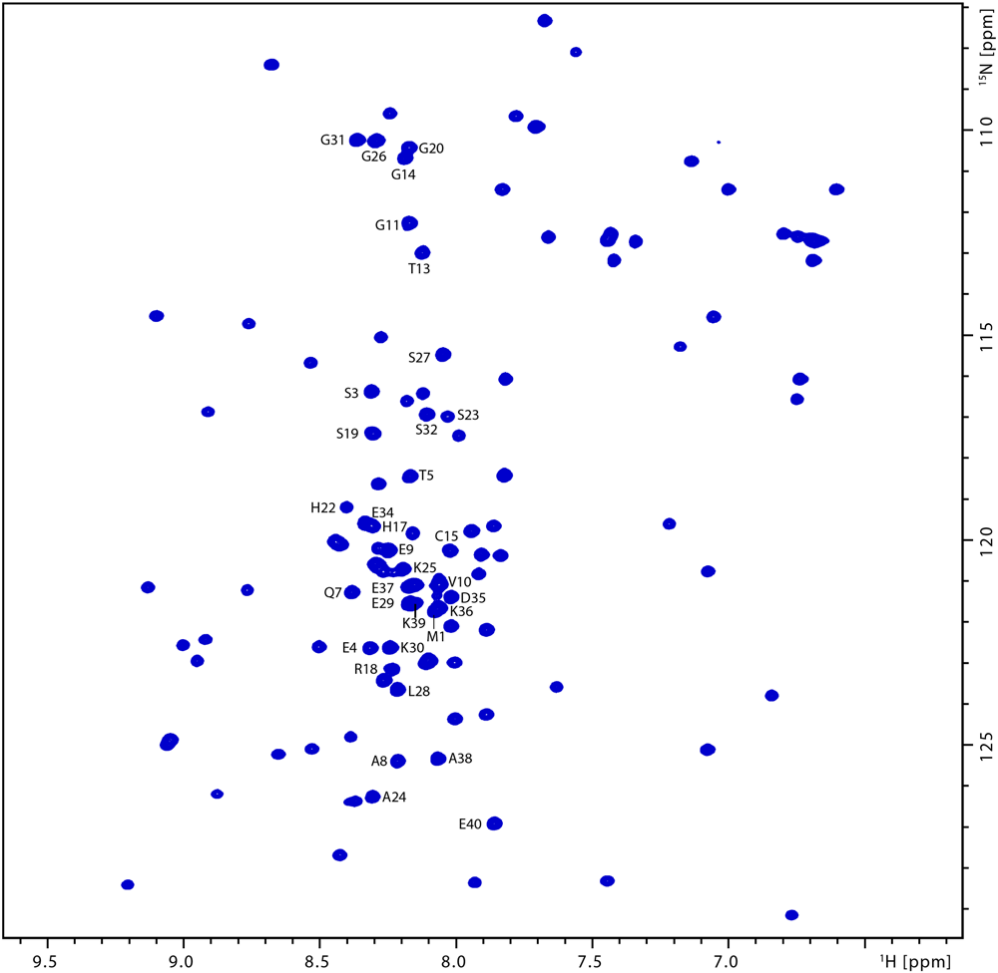
[^1^H,^15^N]-HSQC spectrum of the _GB1_CBS(1–40) fusion protein. Cross peak assignments for the forty _GB1_CBS(1–40) peptide residues are indicated.

Sequence specific resonance assignments for _GB1_CBS(1–40) (Supplementary Table 1) were derived by a set of triple resonance 3D NMR spectra e.g. including well established experiments such as HNCA, HNCOCA, HNCO, HNCACO, HNCACB and HNN. Figure 4 shows the [^1^H,^15^N]-HSQC spectrum of the _GB1_CBS(1–40) fusion protein including the assignments for the 40 N-terminal residues of CBS. The INEPT-based HNN experiment employed in this study essentially leads to the filtering out of signals from the structured part of the GB1 fusion protein due to significant relaxation losses, as shown in Supplementary Figure S1 which gives the [^1^H,^15^N]-projections from the 3D HNCA and HNN experiments. This considerably simplified the data analysis of the _GB1_CBS(1–40) fusion protein. Representative strips indicating the sequential connectivities established are given in the Supplementary Material (Supplementary Figure S2). The calculation of the chemical shift index based on the obtained resonance assignment indicates an intrinsically disordered peptide which is supported by the prediction of the web server (IUPRED^53^) using the primary sequence as sole input.

### NMR study of the heme interaction with the CBS N-terminal peptide (1–40)

**Figure 5:**
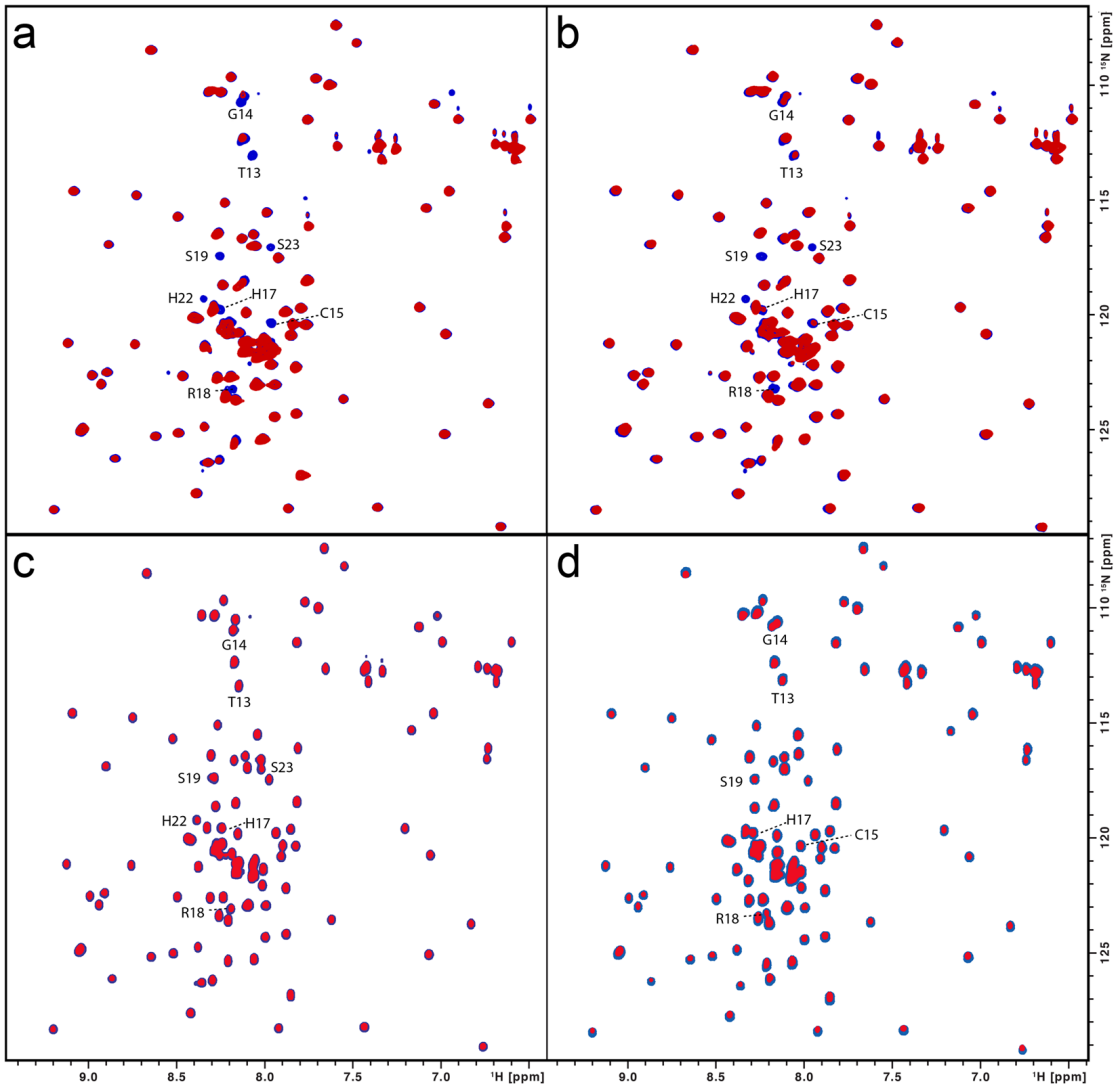
(**a**) Superimposition of the [^1^H,^15^N]-HSQC spectra of the _GB1_CBS(1–40) fusion protein (100 μM) without (blue) and with hemin (50 μM; red). Interacting residues are indicated. (**b**) Superimposition of the [^1^H,^15^N]-HSQC spectra of the _GB1_CBS(1–40) fusion protein (100 μM) without (blue) and with Ga-PPIX (100 μM; red). Interacting residues are indicated. (**c**) Superimposition of the [^1^H,^15^N]-HSQC spectra of the C15S mutant fusion protein (100 μM) without (blue) and with hemin (50 μM; red). (**d**) Superimposition of the [^1^H,^15^N]-HSQC spectra of the H22L mutant fusion protein (100 μM) without (blue) and with hemin (50 μM; red).

**Figure 6:**
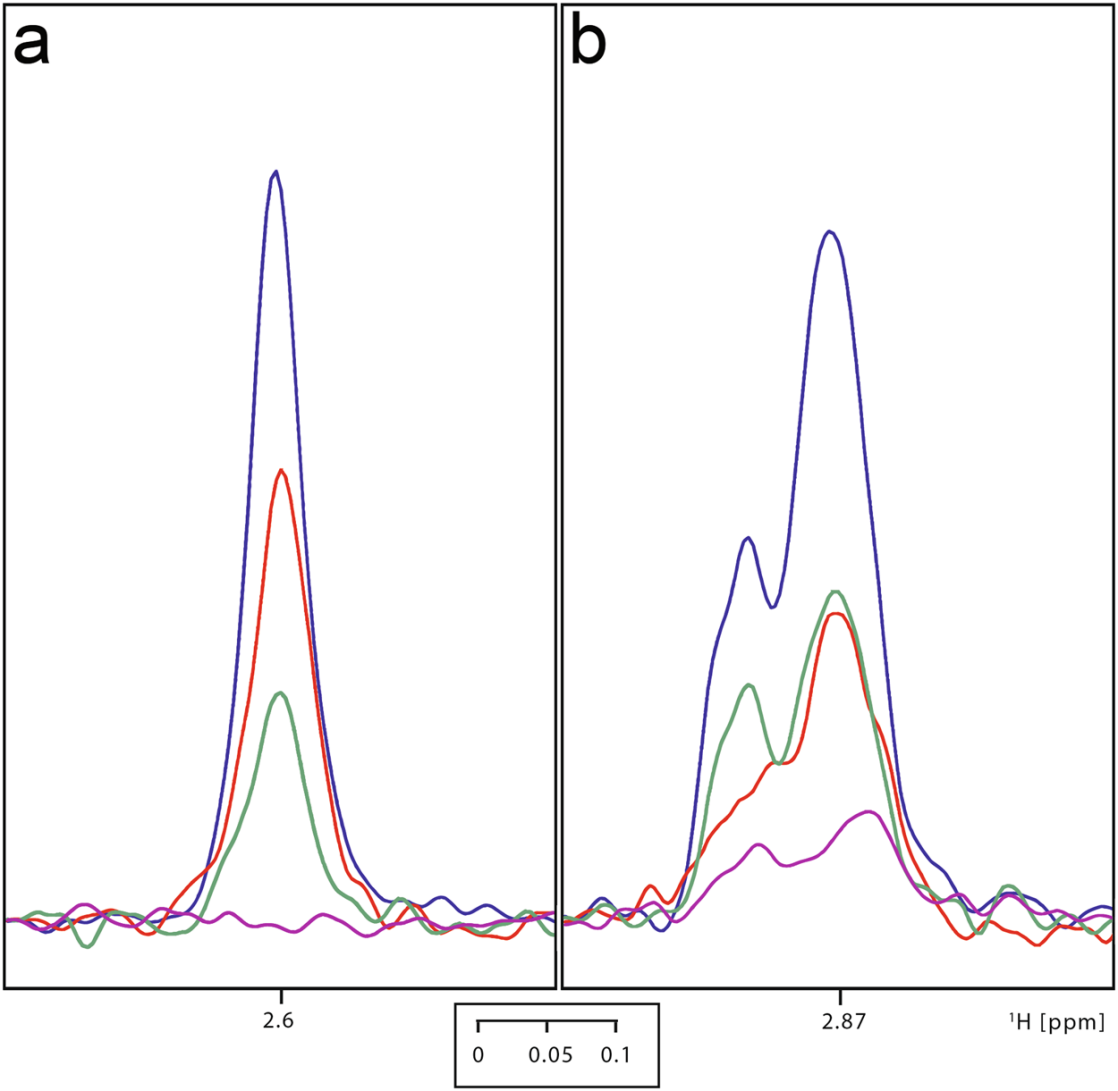
Superimposition of the spectral cross sections from [^1^H,^13^C]-HSQC spectra of _GB1_CBS(1–40) (100 μM) taken at C^β^ positions corresponding to the C15 (**a**) and H17/H22 (**b**) residues in the free (blue), Ga-PPIX (100 μM; red), Fe-PPIX (50 μM; green) and Fe-PPIX (100 μM; pink) exposed states.

Employing 100 μM samples of ^15^N, ^15^N and ^13^C-labelled GB1-fusion peptides, [^1^H,^15^N]-HSQC ligand titration experiments were first carried out using paramagnetic heme samples. As the ligand concentration was varied over a range of 0 to 100 μM, neither chemical shift changes were observed nor new additional peaks with increasing intensities were seen as the concentration of the heme ligand was increased (Supplementary Figure S3). Only a reduction in the intensities of few peaks (T13, G14, C15, H17, R18, S19, H22, S23) was observed, with the reduction in the intensity being maximal near C15 (Fig. 5a). This indicates the spatial proximity of these residues to the paramagnetic center and is consistent with the observation of the Soret band near 420 nm in the UV/Vis spectra (Fig. 2a) of the heme complex which resembles the situation found for transient heme binding by histidine-containing peptides^46,49^. A Soret band near 420 nm is typically associated with the occurrence of a hexacoordinated heme^21,22^. It is worth mentioning, that as with the paramagnetic Fe-PPIX, similar intensity reductions in the [^1^H,^15^N]-HSQC spectrum were also seen with diamagnetic Ga-PPIX (Fig. 5b). This suggests that in addition to the paramagnetic relaxation enhancement (PRE) effect^54,55^ there is also an exchange contribution to the reduction in the intensities observed with the paramagnetic Fe-PPIX. We also observed that the single mutation C15S (Fig. 5c) essentially leads to no effect on the relative intensities of the cross peaks in the HSQC spectra upon heme addition. This indicates that mutation of this residue leads to no heme binding and is consistent with the UV/Vis data of this mutant which shows no sharp Soret peak (Supplementary Figure S5) either at ~420 nm (hexacoordination) or at ~370 nm (pentacoordination). It is also apparent that C15 is involved in the transient heme interaction forming a hexacoordinated complex with H22 forming the other axial ligand (H17 as second coordination site can be excluded due to steric reasons). The role of both amino acids as axial ligands is further supported by the [^1^H,^13^C]-HSQC spectral data recorded with and without heme (Fig. 6, Supplementary Figure S4). Spectral cross sections taken at the C^β^ positions corresponding to the C15 and H17/H22 residues show a similar variation of the intensity indicating the proximity of these nuclei to the heme central ions. Consistent with this, similar intensity variations in the aromatic HSQC spectral cross sections taken at the histidine ^13^C^ε^ and ^13^C^δ^ positions (Supplementary Figure S6) were also observed.

In addition to the C15 mutation we have also mutated the H22 site. Although the UV/Vis spectra of this H22L mutant (Supplementary Figure S5) show a Soret peak at ~369 nm, possibly corresponding to pentacoordination at C15, the [^1^H,^15^N]-HSQC spectra carried out without and in presence of heme display no significant intensity variations of the cross peaks (Fig. 5d). This implies that either the heme-peptide interaction is much weaker and/or the rate of exchange between the heme-bound and the free peptide is not in the favorable range on the PRE timescale^54,55^.

Viewed in totality, the results reported here suggest that the wild-type N-terminal CBS peptide upon heme binding undergoes a conformational change to a hexacoordinated complex with cysteine-15 and histidine-22 as ligands that is sparsely populated and is in exchange with a highly populated free peptide.

## Discussion

Heme binding to the N-terminal stretch of CBS was confirmed by a combined UV/Vis and NMR study. In this study we decided for a fusion approach with the streptococcal protein GB1 since it is known to improve the solubility of the target protein without interfering with its fusion partner because of its inert nature. In addition, GB1 allows usage of easy purification protocols and is characterised with respect to structure and chemical shift assignments^45,56,57^. This fusion approach was also selected since the amino acid sequence of GB1 does not contain further cysteine or histidine residues which constitute classical heme-binding residues. In addition, NMR experiments performed solely on the GB1 protein with heme revealed no interaction between the components (data not shown). The results obtained indicate that the resonance assignments of the intrinsically disordered peptide (IDP) can be conveniently carried out in GB1-fusion since the GB1 signals can be essentially filtered out from the IDP signals. Thus, spectral overlap problems can be minimised without the need for unlabelling approaches^58,59^ or ^13^C-detected experiments^34^. NMR spectroscopy revealed that the forty N-terminal residues constitute an intrinsically disordered region of the protein cystathionine-β-synthase. This is in accordance with the observation that all available X-ray studies did not deliver a structural description of this molecular stretch. In addition, this study demonstrates for the first time that the disordered N-terminal region of CBS contributes heme-binding capacities *via* a second binding site, the CP-based motif at cysteine-15 and a histidine residue at position 22. The functional assay reveals that this heme-binding site at cysteine-15 and histidine-22 as second axial heme ligand increases the efficacy of the enzyme by approx. 30%. The intrinsically disordered N-terminal peptide of CBS is thus involved in transient heme interactions leading to a hexacoordinated complex. Transient binding was observed in both situations, with heme carrying the paramagnetic iron center as well as its diamagnetic substitute, Ga-PPIX. This also stresses the utility of both the Fe^3+^/Ga^3+^ compounds in heme binding studies. The results on _GB1_CBS(1–40) also imply the caveat that a complete functional description of molecules has to carefully consider also regions truncated to facilitate crystallisation for X-ray studies which are often intrinsically disordered, highly dynamic protein regions potentially serving as interaction sites^21,51,60^.

Amino acid sequence comparison of the human and naked mole-rat CBS shows a deletion of the second heme-coordinating residue (histidine-22 in the human IDPR) in the latter species which occurred during evolution from a common ancestor. This might be explained by a lower need for CBS efficacy in sulfur metabolism induced by lower H_2_S levels arising from a reduced dietary uptake of methionine in naked mole-rats^61^. As higher H_2_S levels are connected e.g. to cancer^12^, this could be one reason for the cancer resistance and longevity of naked mole-rats. However, human diet is rich in methionine, thus, evolutionary a contribution of a second heme-binding site might have become necessary to achieve the required efficacy for sulfur processing and to prevent pathological conditions. Hence, the second heme-binding site in CBS described in this study might become an attractive target for drug intervention to prevent pathological conditions without losing the complete biological functions of CBS, thus possibly preventing negative effects on the organism as observed for CBS gene deactivating mutations^6,62^.

## Methods

### Protein expression and purification

CBS(1–413) protein was produced based upon the published protocol^44^. The sample of the CBS(1–413)C15S mutant was generated using site-directed primers, expressed, purified as described below for _GB1_CBS(1–40) except no GB1 tag was used. Superdex 75 10/300 GL column (GE Healthcare, Freiburg, Germany) was used for size exclusion chromatography of CBS (1–413) and its mutant. To study the CBS(1–40) region, _GB1_CBS(1–40) was expressed *via* a fusion protein approach with the streptococcal protein GB1. It was cloned in pET28a vector with GB1 domain attached to N-terminal of CBS(1–40) with flexible linker (TEV protease site - ENLYFQG) to avoid proteolytic cleavage of CBS(1–40) and additionally increasing the yield with stability. _GB1_CBS(1–40) was expressed in *E. coli* BL21(DE3) cells which were grown up to an OD_600 nm_ of 0.7 in the LB medium. Cells were pelleted and transferred to M9 media containing ^15^NH_4_Cl and ^13^C_6_-glucose that will result in labelled _GB1_CBS(1–40). Protein expression was induced at OD_600_ of 0.7 by 0.3 mM IPTG for 18 h at 18 °C. Cells were resuspended in lysis buffer (50 mM Tris/HCl, 300 mM NaCl, 5 mM imidazole, 2 mM β-mercaptoethanol (pH 7.5)) followed by french press to lyse the cells and centrifuged at 10000 × g to collect the supernatant cell lysate. Ni-NTA agarose resin was used to capture the His_6_-tagged _GB1_CBS(1–40) protein followed by wash and elution with 10 mM and 250 mM imidazole, respectively. The His-tag was cleaved from the protein in overnight dialysis buffer (20 mM Tris/HCl, 150 mM NaCl, 2 mM DTT (pH 7.5)) by the addition of 5 U/mg of thrombin (Sigma-Aldrich, Taufkirchen, Germany) at 4 °C. The cleaved protein was concentrated to 1 mL using a 3-kDa Sartorius vivaspin filter and injected onto a 16/60 Hiload S75 size exclusion chromatography column (GE Healthcare, Freiburg, Germany) pre-equilibrated with 20 mM Tris/HCl, pH 6.9, 150 mM NaCl, 2 mM DTT to achive the highest purity. The fractions containing the _GB1_CBS(1–40) were pooled together, concentrated, buffer exchanged into 20 mM sodium phosphate buffer, pH 6.9 and the concentration measured at OD_280_ using UV/Vis spectroscopy. Samples were lyophilised or directly used for the biophysical and NMR experiments.

### UV/Vis spectroscopy

A Multiskan GO microplate spectro-photometer (Thermo Scientific, Dreieich, Germany) was employed for UV/Vis measurements. The hemin solution was prepared by dissolving 1 mM hemin in 30 mM NaOH followed by incubation in the dark for 30 minutes on ice. Subsequently, the hemin solution was diluted with the respective buffer to the concentrations used. _GB1_CBS(1–40) (5 µM) protein was incubated in the dark with different concentration of hemin (0.2–30 µM) for 60 minutes before measuring at 300–600 nm. HEPES-buffer (100 mM, pH 7.0) was used for dissolving protein and hemin.

### Enzyme activity assay and protein concentration

The enzyme activity assay was performed on CBS(1–413) and the CBS(1–413)C15S mutant using established protocols^10^. Fluorescence was read at 450 nm with excitation at 365 nm using an Infinite M1000 microplate reader (Tecan, Männedorf, Switzerland). The protein concentration was determined by UV/Vis spectroscopy.

### NMR spectroscopy

Solution NMR experiments were performed on a Bruker Avance III spectrometers with proton frequencies of 600 MHz at a temperature of 283 K. Data were acquired, processed and analyzed with Topspin (Bruker, Rheinstetten, Germany). The resonance assignment was performed using a combination of experiments such as HNN, HNCO/HNCACO, HNCA/HNCOCA, HCN, HBCBCGCDHD, HNCACB, HBHANH, [^1^H,^15^N]-HSQC, [^1^H,^13^C]-HSQC etc. experiments with _GB1_CBS(1–40) dissolved at concentrations of 700 µM. [^1^H,^13^C]-HSQC spectra of the _GB1_CBS(1–40) were recorded in the free, Ga(III)-protoporphyrin IX (Ga-PPIX) and hemin (Fe-PPIX) exposed state at concentrations of 100 μM and 50 µM, respectively. Ga(III)-protoporphyrin IX chloride and hemin were used as obtained from Frontier Scientific (Logan, USA). Unless otherwise stated, the _GB1_CBS(1–40) and CBS samples were dissolved in 20 mM phosphate buffer and 5% D_2_O.

### Third-party methods

IUPred. Version 1.0 with default parameters was used.

### Data availability

The authors declare that all data supporting the findings of this study are available within the article and its Supplementary Information files, or are available from the corresponding author upon request. The chemical shift assignments of CBS(1-40) have been deposited in the Biological Magnetic Resonance Data Bank (BMRB) under accession code 27351.

## Electronic supplementary material

Supplementary Information
