# Supplementary material for: Heme interaction of the intrinsically disordered N-terminal peptide segment of human cystathionine-β-synthase
Source: Sci Rep. 2018 Feb 6;8:2474. doi: 10.1038/s41598-018-20841-z (PMC5802807; doi:10.1038/s41598-018-20841-z)
Supplement: Supplementary file 1 — Supplementary Information [file 41598_2018_20841_MOESM1_ESM.pdf]

Supplementary Information

Heme interaction of the intrinsically disordered N-terminal peptide segment of human cystathionine-β-synthase

Amit Kumar[1], Amelie Wißbrock[2], Nishit Goradia[1], Peter Bellstedt[3], Ramadurai Ramachandran[1], Diana Imhof[2*] and Oliver Ohlenschläger[1*]

[1]Leibniz Institute on Aging – Fritz Lipmann Institute, Beutenbergstr. 11, D-07745 Jena, Germany.
[2]Pharmaceutical Biochemistry and Bioanalytics, Pharmaceutical Institute, University of Bonn, An der Immenburg 4, D-53121 Bonn, Germany.
[3]Friedrich Schiller University, Faculty of Chemistry and Earth Sciences, Humboldtstr. 10, D-07743 Jena, Germany.

*Correspondence and requests for materials should be addressed to O.O. (email: oliver.ohlenschlaeger@leibniz-fli.de) or D.I. (email: dimhof@uni-bonn.de)

Supporting information contains 6 Figures (S1-S6), and 1 Table (S1).

Figure S1. Superposition of the [^{1}H,^{15}N]-projection from the 3D HNN experiment of the $_{GB1}$CBS(1-40) fusion protein (red) with the HNCA spectrum (blue). Cross peaks arising from the linker between GB1 and CBS(1-40) are annotated.

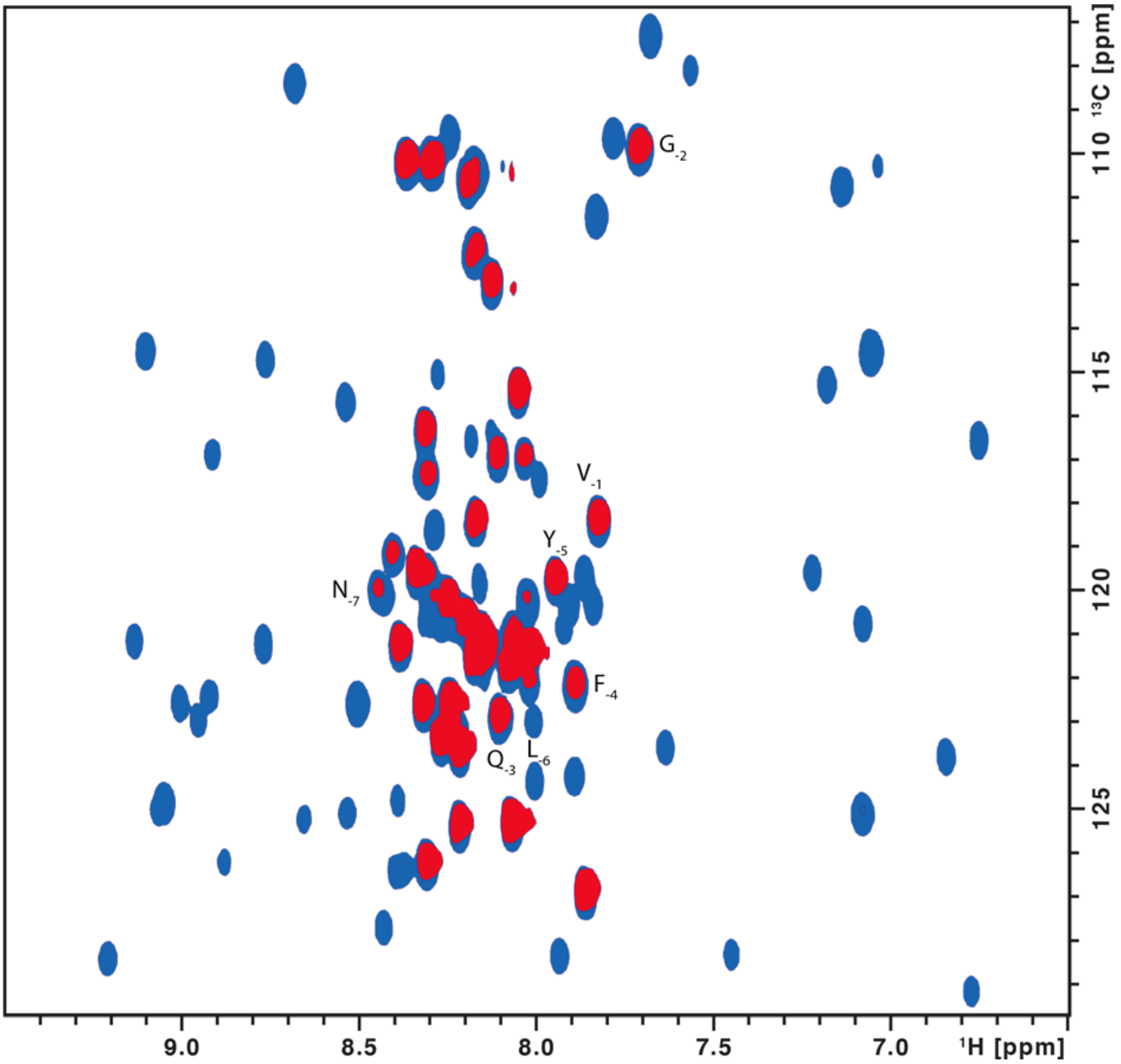

Figure S2. Representative strips from the HNN spectrum of $_{GB1}$CBS(1-40). The sequential connectivities are indicated by arrows.

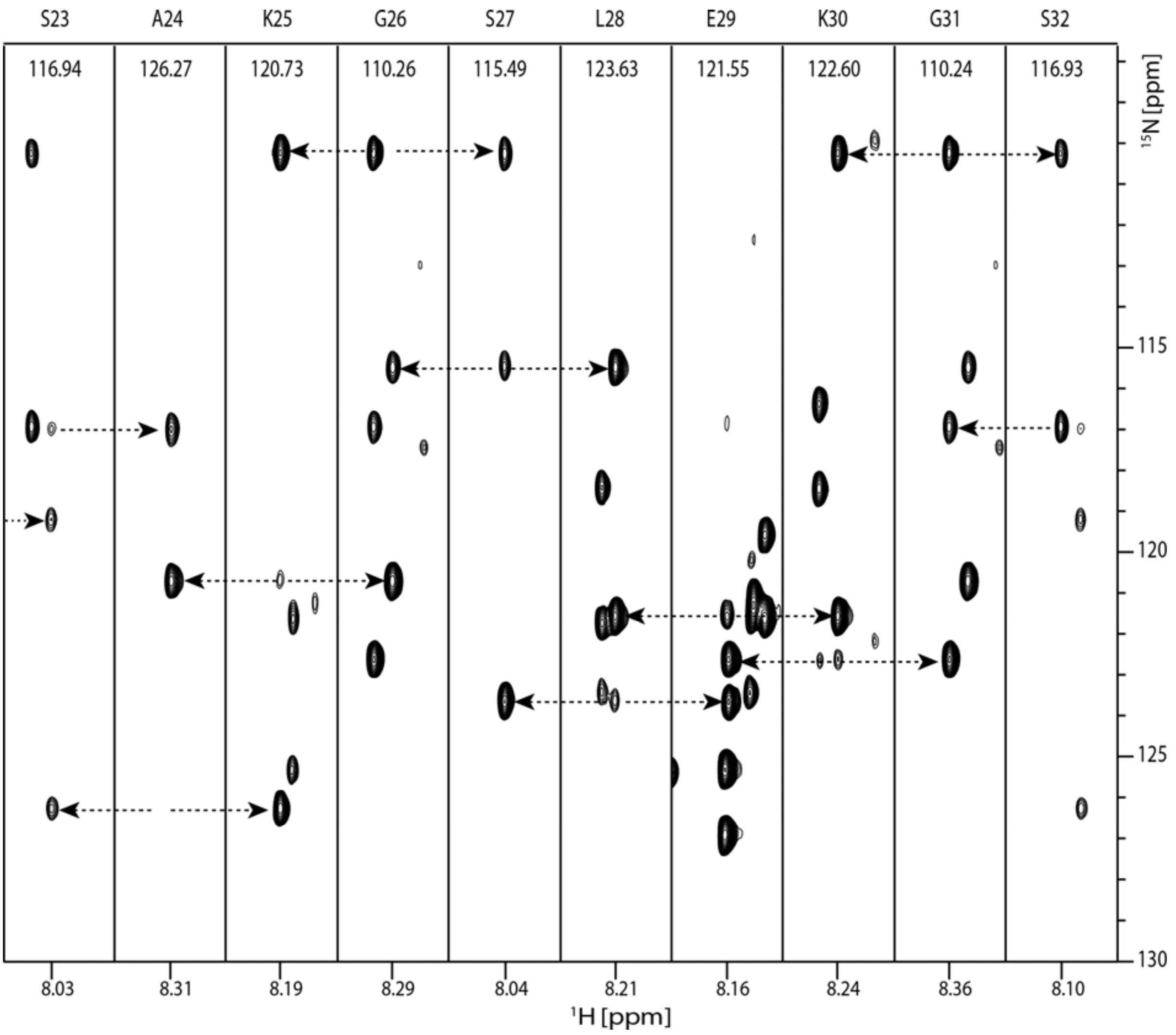

Figure S3A. Spectral cross sections from the [^{1}H,^{15}N]-HSQC spectra of the $_{GB1}$CBS(1-40) fusion protein (100 μM) without and with hemin taken at ^{15}N chemical shift positions corresponding to the amino acid residues indicated (blue – no hemin; red – Ga-PPIX, green – Fe-PPIX). The hemin and Ga-PPIX concentrations used are indicated at the bottom of the panels.

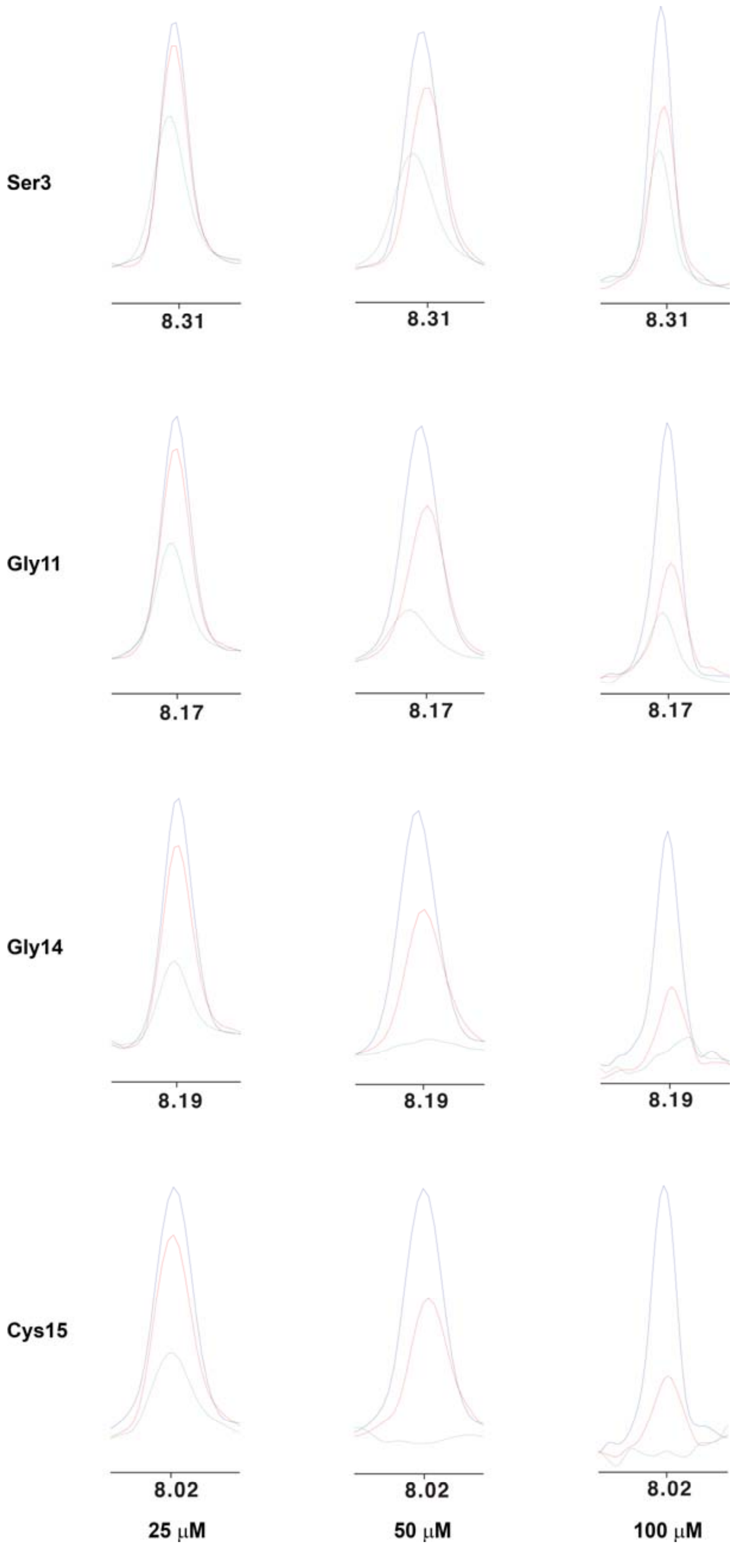

Figure S3B. Spectral cross sections from the [^{1}H,^{15}N]-HSQC spectra of the $_{GB1}$CBS(1-40) fusion protein (100 μM) without and with hemin taken at ^{15}N chemical shift positions corresponding to the amino acid residues indicated (blue – no hemin; red – Ga-PPIX, green – Fe-PPIX). The hemin and Ga-PPIX concentrations used are indicated at the bottom of the panels.

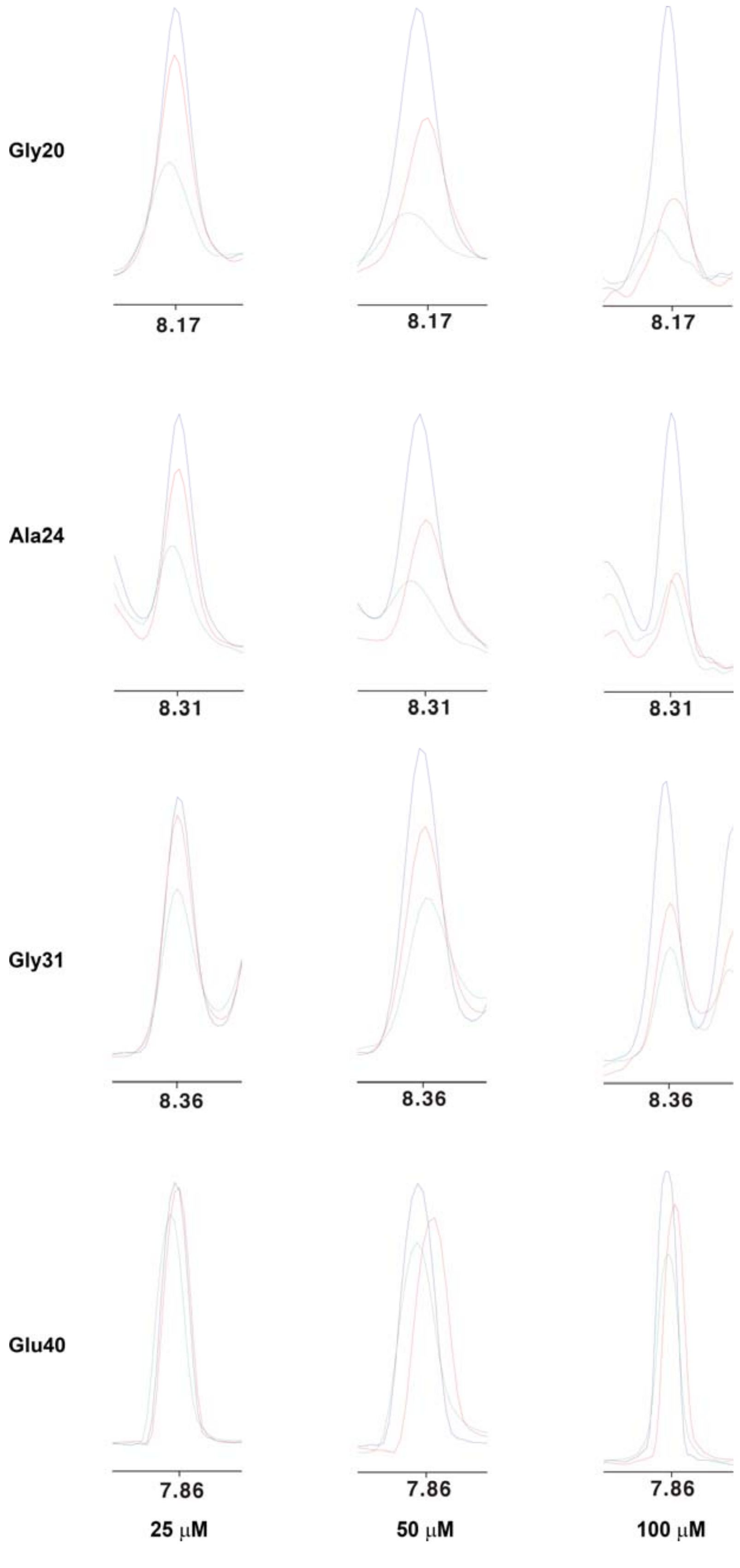

Figure S4. Constant-time [^{1}H,^{13}C]-HSQC spectrum (zoomed plot) of $_{GB1}$CBS(1-40) without heme.

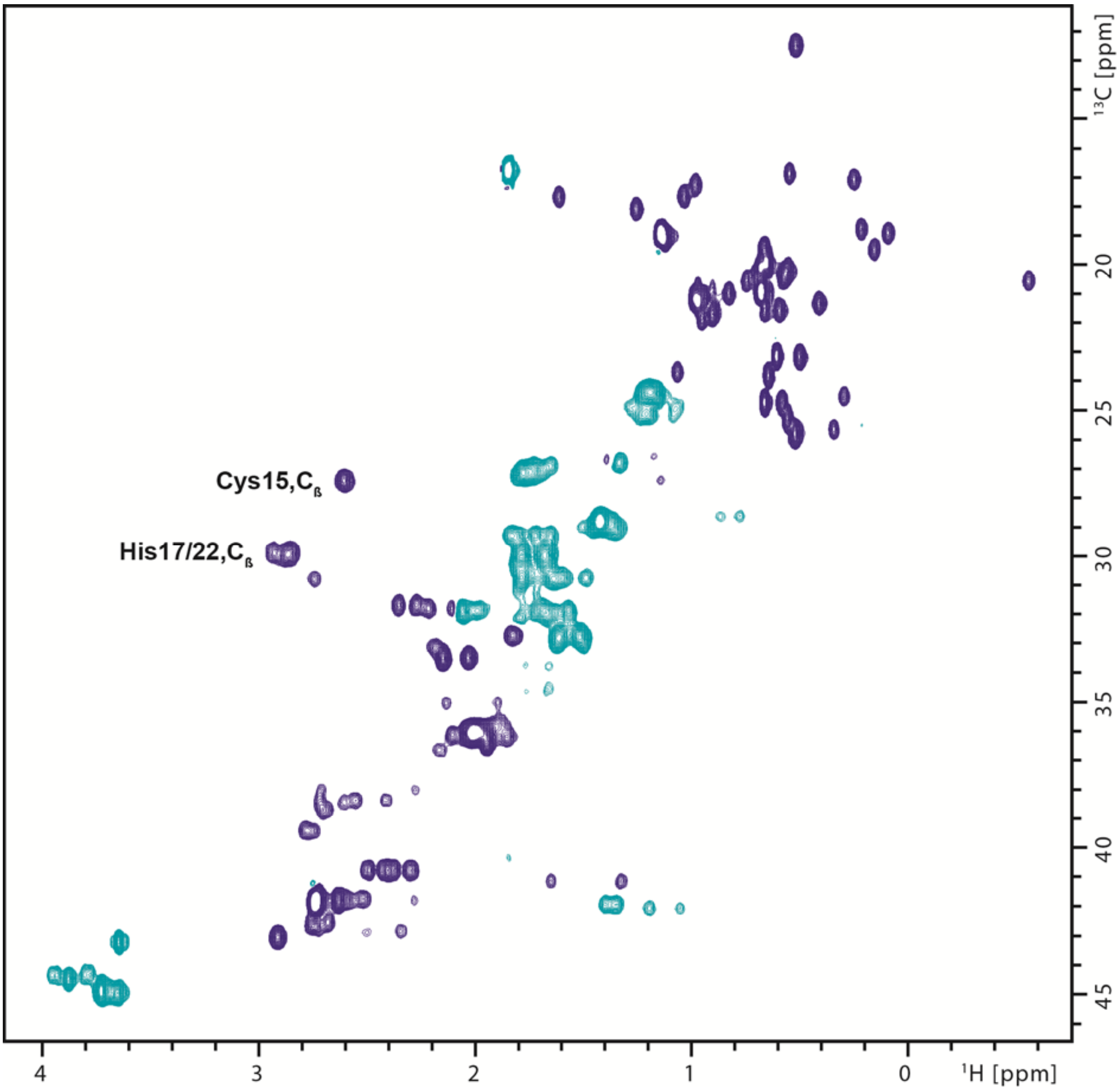

Figure S5. UV/Vis spectra for the $_{GB1}$CBS(1-40)C15S (A) and $_{GB1}$CBS(1-40)H22L (B) mutants. The fusion protein (20 μM protein) was incubated with hemin at concentrations of 2 (blue), 10 (red) and 15 μM hemin (green).

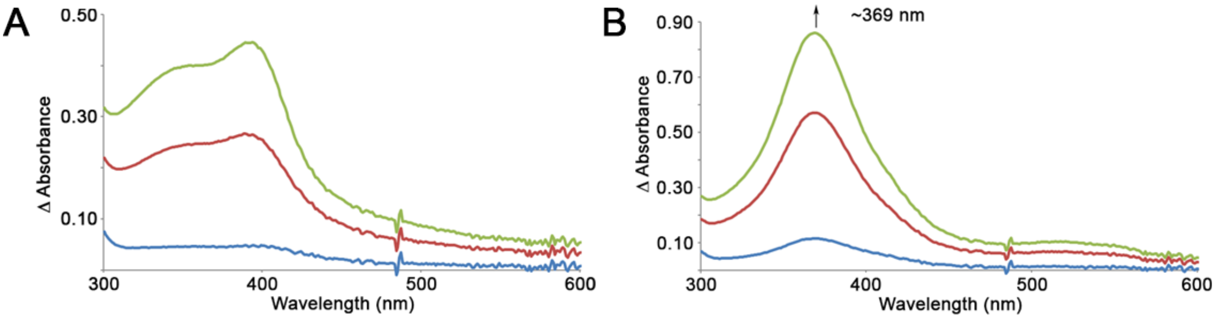

Figure S6. 2D Chemical shift correlation spectra (Figure S6A and S6B) generated via a 3D HCN experiment indicating the ($^{13}C^{\delta}$,$^{1}H^{\delta}$) and ($^{13}C^{\varepsilon}$,$^{1}H^{\varepsilon}$) cross-peaks of His17 and His22 of CBS40. ^{1}H cross-sections taken at the $^{13}C^{\delta}$ and $^{13}C^{\varepsilon}$ positions (cf. Fig. S6B 1-4) in the aromatic [^{1}H,^{13}C]-HSQC spectra generated without (blue) and with (red) the addition of hemin (Fe^{3+}) are given in S6C. Cross peaks 1 and 4 in Fig. S6B/C arise from the thrombin site at the N-terminus of GB1 and serve as indicator for the general solvent PRE effect in Fig. S6C.

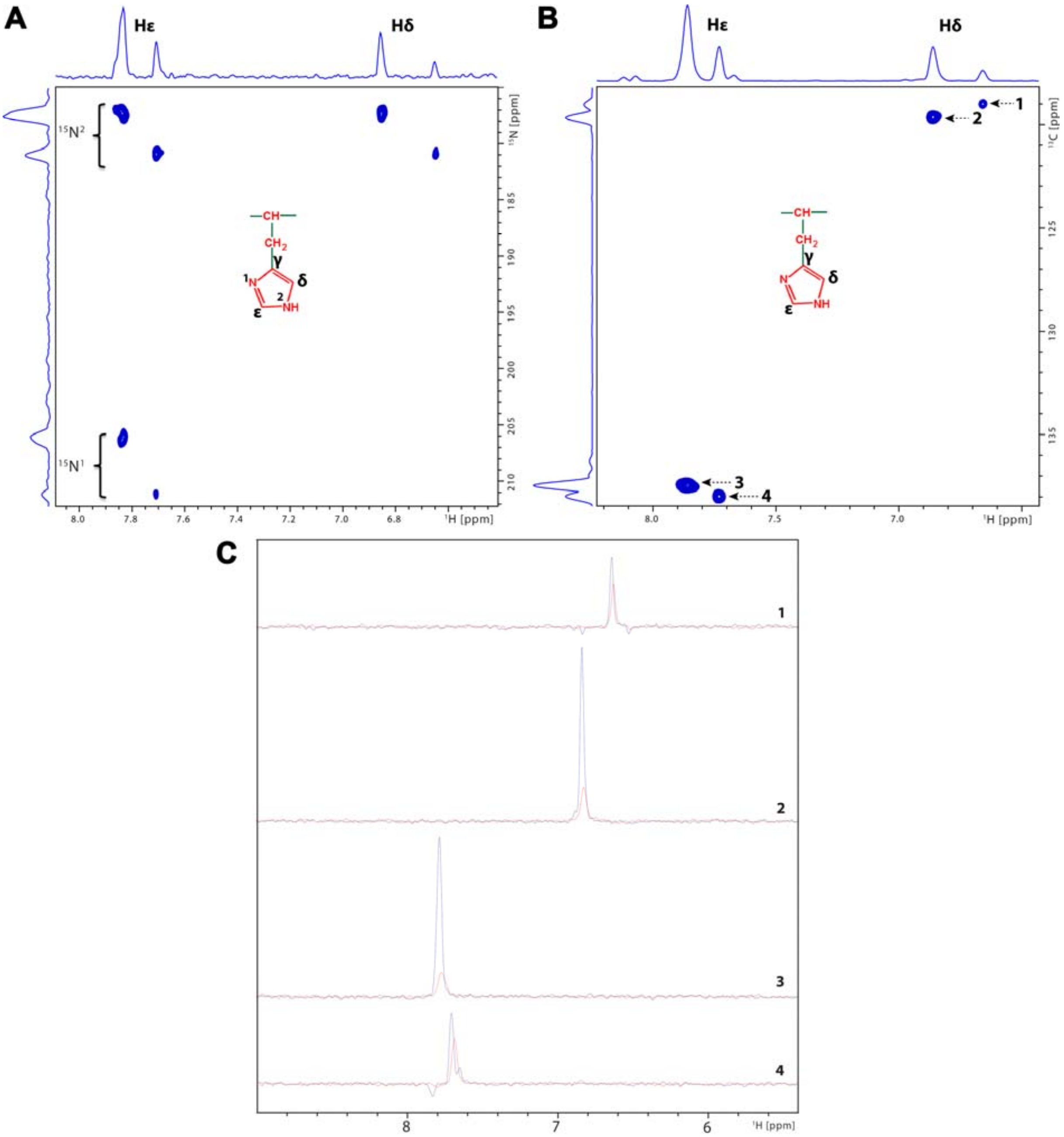

Table S1. Chemical shift assignment of $_{GB1}$CBS(1-40)

		H'	N'	H^{α}	H^{β}	C'	C^{α}	C^{β}	C^{γ}	C^{δ}	C^{ε}
-8	Glu	8.50	122.60	3.97	1.70	175.43	56.42	30.28	36.05	-	-
-7	Asn	8.44	120.05	4.37	2.40,2.55	174.72	52.86	38.26	-	-	-
-6	Leu	8.01	122.10	3.94	1.05,1.20	176.79	55.32	41.86	26.50	24.53,23.40	-
-5	Tyr	7.94	119.77	4.27	2.60,2.71	175.16	57.48	38.41	-	-	-
-4	Phe	7.88	122.19	4.26	2.79,2.75	174.99	57.73	39.38	-	-	-
-3	Gln	8.10	122.95	3.99	1.80,1.64	175.65	55.74	29.23	-	-	-
-2	Gly	7.71	109.91	3.65	-	173.62	44.99	-	-	-	-
-1	Val	7.82	118.42	3.90	1.83	175.52	61.68	32.62	20.81,19.86	-	-
0	Asp	8.26	123.42	4.33	2.41,2.30	175.45	54.17	40.77	-	-	-
1	Met	8.07	121.70	4.53	1.64	-	52.80	32.73	-	-	-
2	Pro	-	-	3.39	1.66,2.06	176.74	64.89	31.82	27.20	50.43	-
3	Ser	8.31	116.39	4.16	3.61	174.40	58.07	63.58	-	-	-
4	Glu	8.31	122.63	4.12	1.67,1.80	176.12	56.09	30.06	36.04	-	-
5	Thr	8.17	118.44	4.31	3.85	-	59.79	69.45	-	-	-
6	Pro	-	-	4.16	1.62,2.05	176.71	63.19	31.92	27.22	50.91	-
7	Gln	8.38	121.25	3.99	1.83,1.70	175.61	55.66	29.22	33.75	-	-
8	Ala	8.21	125.41	4.03	1.12	177.44	52.31	19.04	-	-	-
9	Glu	8.24	120.22	4.05	1.78,1.67	176.22	56.03	30.07	35.97	-	-
10	Val	8.06	121.07	3.91	1.81	176.13	61.78	32.63	19.75,20.71	-	-
11	Gly	8.17	112.27	3.79	-	-	44.38	-	-	-	-
12	Pro	-	-	4.24	1.72,2.05	177.50	63.25	31.85	26.96	49.62	-
13	Thr	8.12	113.00	4.11	4.01	175.10	61.71	69.35	21.24	-	-
14	Gly	8.19	110.69	3.70	-	173.43	44.93	-	-	-	-
15	Cys	8.02	120.25	4.53	2.61	-	56.12	27.32	-	-	-
16	Pro	-	-	4.15	1.57,1.99	176.40	63.11	31.81	27.03	50.58	-
17	His	8.30	119.68	4.37	2.87	174.90	55.91	30.05	-	-	-
18	Arg	8.23	123.16	4.12	1.49	175.81	55.92	30.78	26.79	43.12	-
19	Ser	8.30	117.39	4.26	3.62	174.27	58.01	63.78	-	-	-
20	Gly	8.17	110.42	3.88	-	-	44.60	-	-	-	-
21	Pro	-	-	4.15	1.56,1.97	176.92	63.35	31.70	26.81	49.48	-
22	His	8.40	119.19	4.42	2.94,2.87	174.98	56.01	29.94	-	-	-
23	Ser	8.03	116.98	4.14	3.56	174.01	58.07	63.62	-	-	-
24	Ala	8.31	126.25	4.09	1.14	177.68	52.37	18.94	-	-	-
25	Lys	8.19	120.71	4.03	1.53,1.60	177.11	56.31	32.64	24.44	28.84	41.78
26	Gly	8.29	110.26	3.72	-	173.99	44.98	-	-	-	-
27	Ser	8.04	115.48	4.17	3.61	174.59	58.16	63.52	-	-	-
28	Leu	8.21	123.64	4.11	1.40,1.36	177.26	55.02	41.85	26.62	24.47,23.36	-
29	Glu	8.16	121.55	3.99	1.64,1.77	176.34	56.20	29.89	35.88	-	-
30	Lys	8.24	122.62	4.04	1.52,1.62	176.96	56.12	32.72	24.42	28.73	41.87
31	Gly	8.36	110.24	3.73	-	173.54	44.86	-	-	-	-
32	Ser	8.10	116.93	4.55	3.64	-	56.14	63.19	-	-	-
33	Pro	-	-	4.16	2.06	177.06	63.30	31.71	27.19	50.34	-
34	Glu	8.33	119.57	3.95	1.78,1.67	176.23	56.63	29.87	36.25	-	-
35	Asp	8.01	121.38	4.32	2.38,2.50	176.16	54.23	40.78	-	-	-
36	Lys	8.06	121.63	4.03	1.63,1.51	176.52	56.15	32.60	24.34	28.71	41.73
37	Glu	8.15	121.07	3.98	1.78,1.68	176.00	56.12	29.84	36.07	-	-
38	Ala	8.06	125.34	4.04	1.13	177.29	52.24	18.85	-	-	-
39	Lys	8.17	121.55	4.09	1.62,1.50	175.51	55.85	33.00	24.43	28.78	42.07
40	Glu	7.86	126.91	3.85	1.78,1.64	-	57.75	30.71	-	-	-
